# Did FDA Decisionmaking Affect Anti-Psychotic Drug Prescribing in Children?: A Time-Trend Analysis

**DOI:** 10.1371/journal.pone.0152195

**Published:** 2016-03-31

**Authors:** Bo Wang, Jessica M. Franklin, Wesley Eddings, Joan Landon, Aaron S. Kesselheim

**Affiliations:** 1 Program On Regulation, Therapeutics, And Law (PORTAL), Division of Pharmacoepidemiology and Pharmacoeconomics, Department of Medicine, Brigham and Women’s Hospital and Harvard Medical School, Boston, Massachusetts, United States of America; 2 Harvard Medical School, Boston, Massachusetts, United States of America; 3 Division of Pharmacoepidemiology and Pharmacoeconomics, Department of Medicine, Brigham and Women’s Hospital and Harvard Medical School, Boston, Massachusetts, United States of America; Royal College of Surgeons, IRELAND

## Abstract

**Background:**

Following Food and Drug Administration (FDA) approval, many drugs are prescribed for non-FDA-approved (“off-label”) uses. If substantial evidence supports the efficacy and safety of off-label indications, manufacturers can pursue formal FDA approval through supplemental new drug applications (sNDAs). We evaluated the effect of FDA determinations on pediatric sNDAs for antipsychotic drugs on prescribing of these products in children.

**Methods:**

Retrospective, segmented time-series analysis using new prescription claims during 2003–2012 for three atypical antipsychotics (olanzapine, quetiapine, ziprasidone). FDA approved the sNDAs for pediatric use of olanzapine and quetiapine in December 2009, but did not approve the sNDA for pediatric use of ziprasidone.

**Results:**

During the months before FDA approval of its pediatric sNDA, new prescriptions of olanzapine decreased for both children and adults. After FDA approval, the increase in prescribing trends was similar for both age groups (P = 0.47 for schizophrenia and bipolar disorder; P = 0.37 for other indications). Comparable decreases in use of quetiapine were observed between pediatrics and adults following FDA approval of its pediatric sNDA (P = 0.88; P = 0.63). Prescribing of ziprasidone decreased similarly for pediatric and adult patients after FDA non-approval of its pediatric sNDA (P = 0.61; P = 0.79).

**Conclusions:**

The FDA’s sNDA determinations relating to use of antipsychotics in children did not result in changes in use that favored the approved sNDAs and disfavored the unapproved sNDA. Improved communication may help translate the agency’s expert judgments to clinical practice.

## Background

The Food and Drug Administration (FDA) approves new prescription drugs after an intensive months-long process in which teams of reviewers assess whether the benefits of the drugs outweigh their risks for the clinical conditions in which they have been studied. Once a drug has been FDA-approved, physicians can legally prescribe the drug for any purpose [1–3]. Although these unapproved, or “off-label,” uses of drugs initially lack evidence, some may subsequently gain convincing supporting data through post-approval clinical trials or observational research. Other off-label uses never show adequate documented evidence of efficacy, but continue to be prescribed, subjecting patients to risks of serious adverse events not offset by meaningful benefit [2, 4].

Off-label use of drugs in children and adolescents is especially common because clinical trials are often not performed in pediatric populations prior to initial regulatory approval table [5, 6]. Off-label prescribing of antipsychotic medications among children and adolescents has been controversial due to the uncertain evidence supporting their efficacy in this age group, as well as reports of the increased susceptibility among children and adolescents to adverse effects from these drug products, including endocrine and metabolic abnormalities [7, 8].

If an FDA-approved prescription drug subsequently shows promising clinical effectiveness for an off-label indication, the drug’s manufacturer may file a supplemental application to have the indication authorized by the FDA. The FDA reviews supplemental applications using the same evidentiary standards as new drug applications, and rejects the supplemental applications when the data do not support the proposed indication. Incentives for drug companies to pursue supplemental approvals include limited periods of market exclusivity for the new uses as well as the right to promote the new uses to prescribers and patients (off-label promotion by manufacturers is currently illegal). From 2005–2011, new pediatric indications comprised nearly one-third of efficacy-related supplemental approvals [9].

Do FDA decisions on supplemental applications affect prescribing practices for the off-label use under review? Previous studies have shown variable effects of FDA interventions on patients’ health care utilization or physicians’ behaviors [10, 11]. To our knowledge, no studies have explored the effect of FDA decisionmaking around supplemental applications on physicians’ prescribing practices. FDA approval of a supplemental indication should result in increased prescribing of the drug for this validated purpose relative to other uses and patient populations not affected by the agency’s decision, and prescribers should turn away from supplemental indications that the FDA rejects. We examined prescribing rates before and after FDA decisionmaking relating to supplemental indication applications submitted for three atypical antipsychotic medications used in pediatric patients: olanzapine (Zyprexa), quetiapine (Seroquel), and ziprasidone (Geodon).

## Methods

### Study Design

In December 2009, the FDA approved sNDAs expanding the validated indications of the atypical antipsychotics olanzapine and quetiapine to include pediatric patients with schizophrenia and bipolar disorder [12, 13]. The FDA simultaneously reviewed a supplemental application related to the atypical antipsychotic ziprasidone for the same indications, but did not approve this application. We conducted a retrospective, time-series analysis using new prescription claims for these three medications from 2003–2012. This study was approved by the Brigham and Women’s Hospital Institutional Review Board. All participants provided informed consent to have their information stored in the Optum Research database, the data source for our study. No informed consent was obtained for the purposes of this study because the data was analyzed anonymously.

### Study Population

The claims data from the Optum Research Database contains medical and pharmacy data on insurance claims for more than 14 million current beneficiaries of the UnitedHealth commercially insured population, and has previously been used for similar analyses in the medical literature [14, 15]. This source population reflects the nationwide geographic distribution of the health insurer, and has demographics similar to the US census age distribution for sex and age groups <65 years (data from Georgia were excluded due to validation concerns).

Claims were aggregated by calendar quarter. A new prescription was defined as the first prescription filled by a patient (a new user) for olanzapine, quetiapine, or ziprasidone with no prior fill for that particular medication in the preceding 180 days. We only included new users who maintained continuous eligibility, had at least one inpatient or outpatient claim, and filled at least one prescription during the prior 180 days to ensure that our data did not include as new users patients who may have filled prescriptions for the drugs elsewhere before enrolling in UnitedHealth. Subjects were allowed to contribute multiple episodes in the time series if they stopped using the medication and restarted use more than 180 days later.

We identified new users of our study drugs who had diagnosis codes within the previous 180 days that corresponded to schizophrenia and bipolar disorder (see S1 Table for ICD-9-CM codes) and divided them into adult vs. pediatric users (≥18 years vs. <18 years). All patients without a diagnosis for psychotic disorder or bipolar disorder were categorized as “other” and also divided by age. Our outcomes of interest for each psychotropic agent were the quarterly incidence of new users for each indication and age group, expressed as new prescriptions per 100,000 active adult or pediatric program enrollees.

### Time intervals

Data collection started in the third quarter of 2003. By that time, our three study drugs had been approved for treatment of schizophrenia in adults and olanzapine had also been approved for treatment of adults with bipolar disorder. In 2004, both quetiapine and ziprasidone were approved for the latter indication. None had any approved indications in pediatric patients.

The first public signal from the FDA related to use of these products in pediatric patients occurred in June 2009 when the FDA’s Psychopharmacologic Drugs Advisory Committee voted strongly in favor of approval of olanzapine and quetiapine for both schizophrenia and bipolar disorder, but provided mixed votes on ziprasidone’s efficacy and safety for bipolar mania in children (12 for, 2 against, 4 abstaining on the question of whether there was sufficient efficacy; 8 for, 1 against, 9 abstaining on the question of whether there was sufficient safety) [16]. In December 2009, the FDA approved olanzapine for use in schizophrenia and bipolar disease in adolescents aged 13–17, and approved quetiapine for schizophrenia in adolescents aged 13–17 and bipolar mania in children aged 10–17. By contrast, ziprasidone was not approved for either indication for children, and remained unapproved by December 2012 (the end of our study period) [17].

Thus, the first time period in our analysis ran from the third quarter of 2003 through the second quarter of 2009. After a skip period that included the third and fourth quarters of 2009 to cover the months following the FDA’s Advisory Committee votes and the FDA’s period of deliberation, our second time period extended from the first quarter of 2010 through the fourth quarter of 2012, the most recent quarter of data available at the time of analysis.

### Statistical analysis

We fit separate Poisson regressions for each drug-indication combination. Each regression modeled the number of new prescriptions over time, with separate trends for adults and children both before and after the second quarter of 2009. For each model there are two observations per quarter, one for adults and one for children. Each model included the number of enrollees as an offset, to account for the denominators of the rates.

The parameter of interest in each model compared the pre-post change in prescribing trend in children with the pre-post change in adults. We used the bootstrap to estimate a standard error and confidence interval for each parameter and test for a statistically significant change. The bootstrap resamples with replacement from the observed data and uses the variation in the resampled datasets to measure the uncertainty in the original analysis. All analyses were done in Stata 13.1 (StataCorp LP, College Station TX).

## Results

### Cohort characteristics

Across all study drugs, a higher proportion of female patients was found in the adult prescription-filling population compared to pediatric patients (Table 1). In addition, the co-morbidity index was low in both age groups. Adult patients prescribed the study drugs had higher numbers of prescription fills, comparable total physician visits, and fewer psychiatric visits compared to pediatric patients in the cohort in the 180 days prior to filling a study drug.

**Table 1:** Demographic characteristics of new users of quetiapine, olanzapine, and ziprasidone in the Optum database, 2003–2012.

	Quetiapine		Olanzapine		Ziprasidone	
Characteristic	Less than 18 years old (n = 21,044)	18 years and older (n = 141,187)	Less than 18 years old (n = 6,471)	18 years and older (n = 58,118)	Less than 18 years old (n = 4,813)	18 years and older (n = 18,893)
Age, mean years (SD)	13.9 (3.3)	43.8 (15.6)	13.4 (3.5)	45.9 (16.4)	13.8 (3.1)	40.6 (13.5)
**Sex, Number (Percent)**						
Male	11,678 (55.5)	54,950 (38.9)	4,395 (67.9)	25,131 (43.2)	2,644 (54.9)	6,014 (31.8)
Female	9,366 (44.5)	86,237 (61.1)	2,076 (32.1)	32,987 (56.8)	2,169 (45.1)	12,879 (68.2)
**Medical History**						
Total prescriptions filled in prior 180 days, mean (SD)	5.1 (3.2)	8.9 (5.7)	5.3 (3.2)	8.6 (5.6)	4.6 (3.1)	7.7 (5.6)
Charlson-Romano comorbidity index, mean (SD)	1.2 (0.6)	1.4 (1.5)	1.3 (0.8)	1.5 (1.7)	1.2 (0.5)	1.2 (1.1)
Total physician visits in prior 180 days, mean (SD)	10.1 (11.5)	11.7 (16.1)	8.7 (12.6)	10.1 (16.5)	10.6 (11.2)	12.0 (14.3)
Total psychiatrist visits in prior 180 days, mean (SD)	5.3 (7.8)	3.6 (6.3)	4.0 (6.5)	2.5 (5.6)	5.8 (7.7)	5.1 (7.4)

SD = standard deviation.

### Trends in olanzapine prescriptions

**Fig 1:**
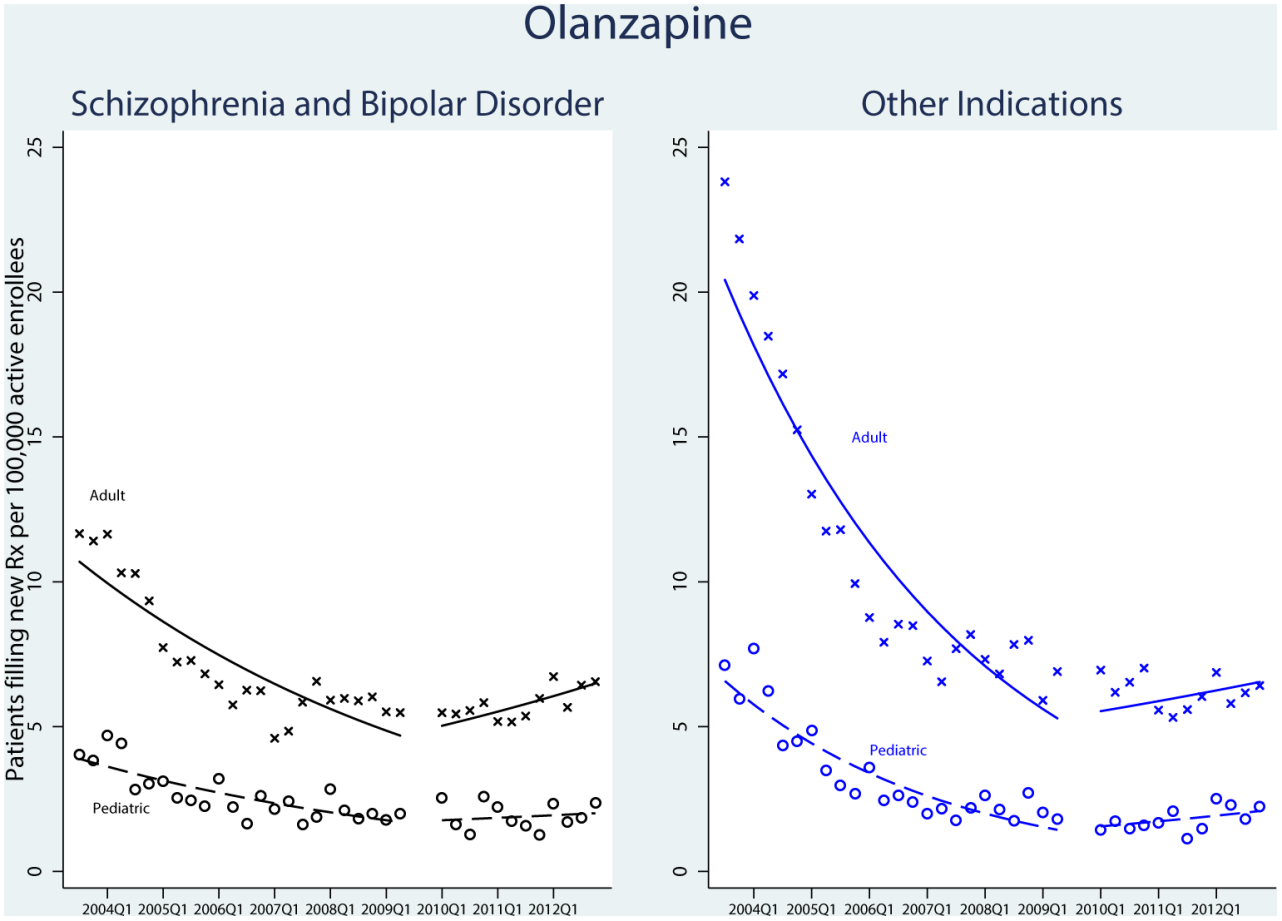
Time-Series Analysis of New Users of Olanzapine, 2003–2012. Patients were enrolled in UnitedHealth insurance. Adults were defined as ≥18 years old and pediatrics defined as <18 years of age. The first time interval ran from the third quarter of 2003 through the second quarter of 2009, when the US Food and Drug Administration’s Advisory Committee convened to discuss use of olanzapine in the pediatric population. The skip period included the third and fourth quarters of 2009 to cover the aftermath of the FDA’s Psychopharmacologic Drugs Advisory Committee votes and the agency’s period of deliberation. The second time period ran from the first quarter of 2010 through the fourth quarter of 2012.

There were 54,352 qualifying olanzapine prescriptions. Among pediatric patients, we observed a decrease in the rate of new prescriptions for the treatment of schizophrenia and bipolar disorder in the years before FDA action, from a high of 4.7 new users per 100,000 active pediatric enrollees in the first quarter of 2004 to a nadir of 1.6 in the third quarter of 2007 (Table 2). Fig 1 shows the utilization trend. There was an increase in the rate of new prescription fills in the second time period. We observed similar trends in the adult population, with no statistically significant difference in the trends between the two age groups (P = 0.47).

**Table 2:** Statistical analyses of utilization trends of olanzapine, quetiapine, and ziprasidone, using Poisson regression models.

					Interaction	
Study Drug	Indication(s)	Age Group	Time Interval	Incidence Rate Ratio (95% CI)	Interaction Effect (95% CI)	P-value
Olanzapine	Schizophrenia/ bipolar	Pediatric	Before	0.96 (0.95, 0.97)	0.99 (0.96, 1.02)	0.47
			After	1.01 (0.99, 1.03)		
		Adult	Before	0.96 (0.96, 0.97)		
			After	1.02 (1.01, 1.04)		
	Other	Pediatric	Before	0.94 (0.92, 0.95)	1.02 (0.98, 1.06)	0.37
			After	1.03 (1.00, 1.05)		
		Adult	Before	0.94 (0.93, 0.95)		
			After	1.02 (1.00, 1.03)		
Quetiapine	Schizophrenia/ bipolar	Pediatric	Before	1.01 (1.00, 1.02)	1.00 (0.97, 1.02)	0.88
			After	0.96 (0.95, 0.98)		
		Adult	Before	1.02 (1.01, 1.03)		
			After	0.98 (0.96, 0.98)		
	Other	Pediatric	Before	1.00 (1.00, 1.01)	0.99 (0.97, 1.02)	0.63
			After	0.96 (0.95, 0.98)		
		Adult	Before	1.01 (1.01, 1.02)		
			After	0.98 (0.97, 0.98)		
Ziprasidone	Schizophrenia/ bipolar	Pediatric	Before	1.02 (1.00, 1.03)	1.01 (0.97, 1.05)	0.61
			After	0.97 (0.95, 0.99)		
		Adult	Before	1.02 (1.01, 1.03)		
			After	0.96 (0.95, 0.97)		
	Other	Pediatric	Before	0.99 (0.98, 1.01)	1.01 (0.97, 1.05)	0.79
			After	0.97 (0.95, 0.98)		
		Adult	Before	1.00 (0.99, 1.01)		
			After	0.97 (0.95, 0.98)		

Each incidence rate ratio compares the rate of new prescriptions in a quarter with the rate in the previous quarter; a ratio less than 1 means that the rate is decreasing. There are separate slopes for the periods before and after the Advisory Committee meeting. The interaction effect is the ratio of the pediatric change (comparing “After” with “Before”) to the adult change. There is one model for each drug-indication combination. Confidence intervals (CIs) were found by the bootstrap BCa (bias-corrected and accelerated) method.

The use of olanzapine for other indications followed a similar trend in the pediatric and adult populations. No statistically significant difference was seen in the fill trend between these age groups (P = 0.37).

### Trends in quetiapine prescriptions

**Fig 2:**
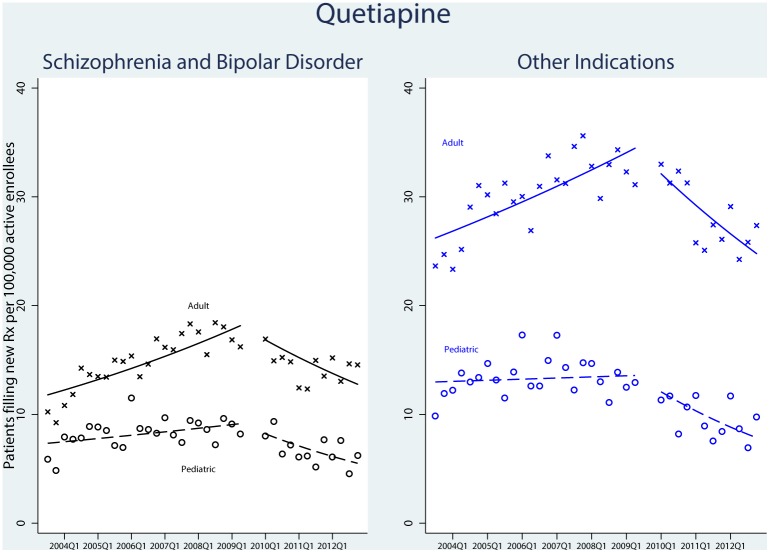
Time-Series Analysis of New Users of Quetiapine, 2003–2012. Patients were enrolled in UnitedHealth insurance. Adults were defined as ≥18 years old and pediatrics defined as <18 years of age. The first time interval ran from the third quarter of 2003 through the second quarter of 2009, when the US Food and Drug Administration’s Advisory Committee convened to discuss use of quetiapine in the pediatric population. The skip period included the third and fourth quarters of 2009 to cover the aftermath of the FDA’s Psychopharmacologic Drugs Advisory Committee votes and the agency’s period of deliberation. The second time period ran from the first quarter of 2010 through the fourth quarter of 2012.

There were 155,223 qualifying prescriptions for quetiapine. Fig 2 shows the new prescription trends for quetiapine. In pediatric patients, the rate of incident prescriptions for the treatment of schizophrenia and bipolar disorder increased during the first time period and decreased after FDA action on the sNDA. There was no statistically significant difference in utilization between the pediatric and adult populations for these indications following sNDA approval (P = 0.88).

For all other indications, there was also no significant difference in the trend of quetiapine prescriptions between the age groups (P = 0.63).

### Trends in ziprasidone prescriptions

**Fig 3:**
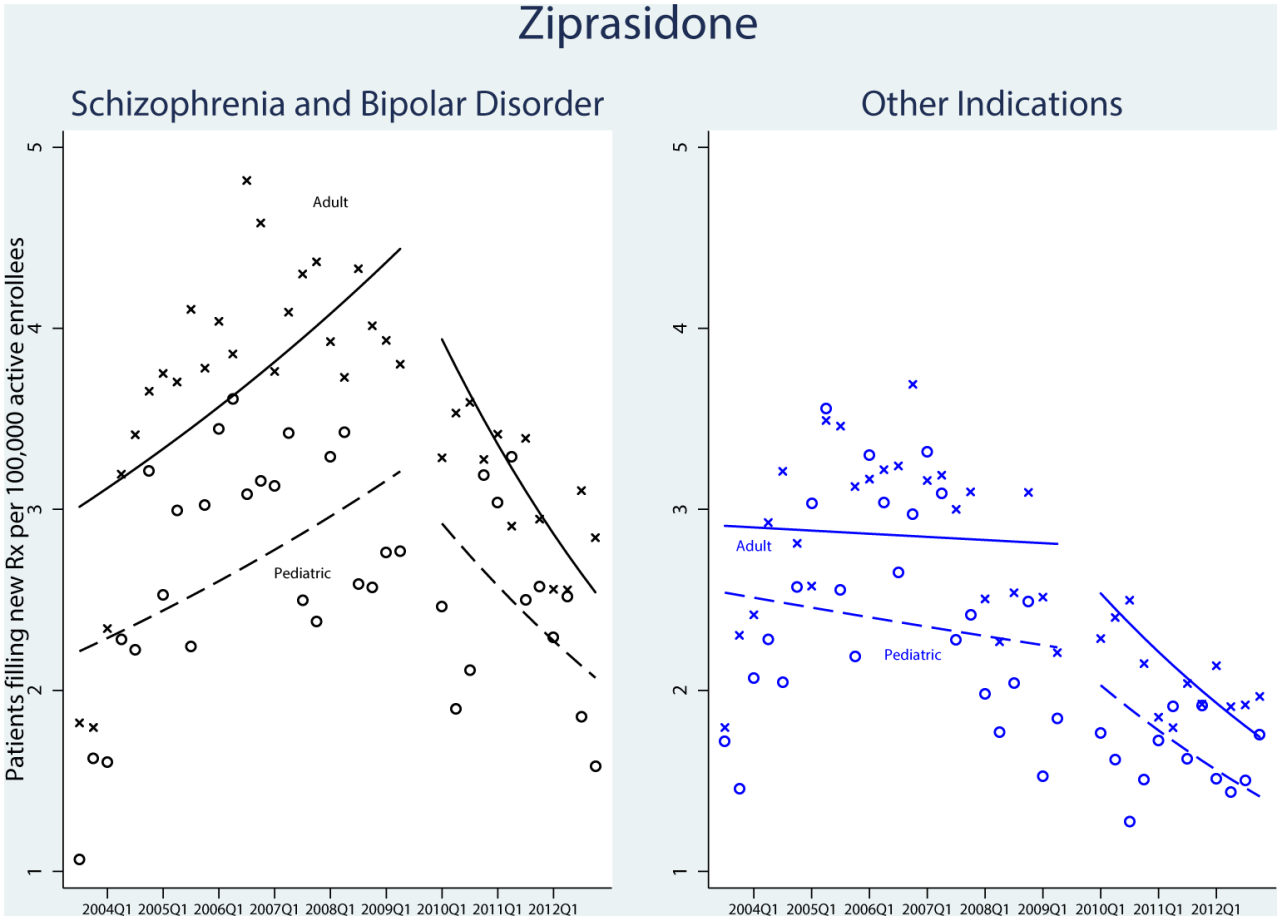
Time-Series Analysis of New Users of Ziprasidone, 2003–2012. Patients were enrolled in UnitedHealth insurance. Adults were defined as ≥18 years old and pediatrics defined as <18 years of age. The first time interval ran from the third quarter of 2003 through the second quarter of 2009, when the US Food and Drug Administration’s Advisory Committee convened to discuss use of ziprasidone in the pediatric population. The skip period included the third and fourth quarters of 2009 to cover the aftermath of the FDA’s Psychopharmacologic Drugs Advisory Committee votes and the agency’s period of deliberation. The second time period ran from the first quarter of 2010 through the fourth quarter of 2012.

There were 23,451 qualifying ziprasidone prescriptions. Fig 3 shows the trend for new ziprasidone prescriptions. Similar to quetiapine, we observed an increase in the rate of new prescriptions for the treatment of schizophrenia and bipolar disorder for children and adolescents during the period leading up to sNDA regulatory review, from 1.1 new users per 100,000 active pediatric enrollees in the third quarter of 2003 to a high of 3.4 in the second quarter of 2006. Following FDA non-approval, the rate of new prescription filling decreased to a low of 1.6 new users per 100,000 active enrollees in the fourth quarter of 2012. There was no statistically significant difference in the trend for these indications compared with the adult population (P = 0.61).

The fill rate of ziprasidone for other indications followed a similar pattern in the adult and pediatric populations, with no significant difference in the utilization trend between the age groups (P = 0.79).

## Discussion

Our time-series analysis revealed no significant differences in utilization trends of olanzapine, quetiapine, or ziprasidone in children compared with adult patients in the time periods before and after FDA determinations on these psychotropics’ supplemental applications relating to their use in children. Prevalence of new users of olanzapine increased slightly in both children and adults, while initiation of quetiapine and ziprasidone decreased.

Given that the FDA is one of the nation’s premier public health authorities and that its scientists re-analyze and weigh the totality of evidence when considering a supplemental application for a drug, its decision in 2009 to authorize the use of olanzapine and quetiapine in children would be expected to result in increases in utilization of the two drugs with approved sNDAs. One mechanism for such a predicted increase would be prescribing for new patients by physicians who were previously uncertain about these agents’ benefit/risk ratios. Another would be switching of pediatric patients previously prescribed ziprasidone—found at the same time to have risks that outweighed its benefits—or another antipsychotic agent off-label to either olanzapine or quetiapine, though physicians often maintain patients on the same medication once they have demonstrated adequate response. Similarly, new use of ziprasidone among children would be expected to decrease as physicians turned away from initiating children on this medication. In particular, the use of all three of these drugs in pediatric patients would be expected to differ significantly from prescribing trends among adults, who were not affected by the FDA’s review of the supplemental applications. However, our study did not show these prescribing trends.

Use of all three psychotropic agents among pediatric patients before the FDA’s Advisory Committee votes in 2009 was high. Prescribing during this time may have been driven by individual clinical judgment of physicians and patients, supported by small, limited studies that signaled favorable outcomes for the use of these drugs in children [18–21]. In addition, all three manufacturers—Eli Lilly (olanzapine), AstraZeneca (quetiapine), and Pfizer (ziprasidone)—engaged in widespread illegal promotion of these agents during this time to enhance their off-label prescribing for many conditions in children [22–24]. The off-label promotion led to government investigations and ultimately settlements with admissions of guilt from all three companies, associated with substantial and widely-publicized civil and criminal fines announced in 2009–2010 (for a full time-line of events, see S1 Fig**)** [25–27]. The initiation and conclusions of these lawsuits may have impacted promotion and prescribing among pediatric patients, counteracting the uptake of olanzapine and quetiapine for schizophrenia and bipolar disease in children and adolescents that might have been predicted by the approval of their sNDAs, which took place around the same time. Studies highlighting the cardiovascular and metabolic adverse effects associated with the use of antipsychotic treatments in the pediatric population [28, 29], also published around the time of the supplemental application decision, likely further attenuated the uptake of these drugs in children and adolescents, as did a growing negative perception of these drugs due to large, well-controlled observational studies linking their use to adverse events like diabetes as well as mortality [30–33]. In addition, two other psychotropic agents—risperidone (Risperdal) and aripiprazole (Abilify)—were approved for treatment of pediatric schizophrenia and bipolar disorder in 2007–2008 [34–36], also moderating uptake of olanzapine and quetiapine in children and adolescents. These outside factors limit our ability to isolate how the FDA’s supplemental approval of two of our study drugs impacted their use among pediatric patients.

However, any contemporaneous social or market forces reducing psychotropic use in children and adolescents would not explain the observed utilization trend for ziprasidone, which did not decrease compared with use among adults following FDA's non-approval. For ziprasidone, lack of physician awareness about the FDA decision may have contributed to our observed trend. FDA approvals of the pediatric sNDAs for both olanzapine and quetiapine were broadcast widely by the manufacturers as well as the FDA through press releases [11, 12] and updated prescription drug labels. By contrast, the FDA’s non-approval decision pertaining to ziprasidone was not acknowledged with any official communication or press release, reflecting the FDA’s default position of keeping confidential anything that could be construed as commercial information. Physicians unaware of the FDA’s deliberations may have continued initiating new pediatric patients on ziprasidone, exposing them to a psychotropic drug that had been determined to have an adverse effect profile which outweighed its potential benefits in this age group. Another factor hindering dissemination of the FDA’s negative judgment regarding ziprasidone’s use in children may have been researchers and journalists who misinterpreted the FDA Advisory Committee’s mixed vote on ziprasidone’s efficacy and safety as signifying official FDA approval of the drug for this use [37, 38].

Enhanced transparency and communication may allow the FDA’s sNDA process to more optimally serve the best interests of the public health. A first step would be to routinely inform prescribers and patients about sNDAs and sBLAs that were not approved through the same communication channels as the FDA informs the medical community about those that were. For example, the FDA could require both approved as well as non-approved indications to be included in the medication’s prescription drug label, similar to how it utilizes the “Limitation of Use” section in the label of recently approved therapeutics to explicitly state the non-indicated uses [39–41]. The label could additionally include a brief rationale supporting the non-approval decision and provide references to records of the studies and clinical data (in the published literature, or on ClinicalTrials.gov) for prescribers seeking to review the evidence. The FDA should also make the underlying aggregated clinical trial data public, as it does in FDA Approval Packages of approved new drug indications. Since drug labels are a widely used resource and also serve as the bases for other frequently-consulted sources of drug information [42], prescribers will be better informed about the agency decisions and be more empowered to promptly translate the FDA reviewers’ expert judgments to clinical practice.

In addition, other pathways for communication about adverse FDA decisions related to common off-label uses of approved drugs should be explored. For example, FDA could create a channel within its MedWatch Safety Alerts database to inform physicians when new non-approval decisions are made pertaining to supplemental applications. These actions would allow the FDA to take another step towards greater transparency, and would follow on the heels of other initiatives the FDA has undertaken to promote greater openness [43–45].

Our study has several limitations. Our data source includes only commercially-insured patients and therefore may not be representative of the pediatric and adult psychotropic use in the overall US population, which has been reported as higher among lower-income patients on government-sponsored Medicaid insurance. Second, while FDA clearly reviewed and did not approve ziprasidone for use in the pediatric population during our study period, we cannot confirm that the FDA rendered a formal judgment for this indication, given the confidentiality that currently surrounds such decisions. Despite the lack of confirmation by the agency, it is highly unlikely that the FDA would have withheld a positive decision on an sNDA over 4 years after it was first submitted in October 2008 [46]. Finally, our finding of no relative change in prescribing of the antipsychotic agents in our study among pediatric populations after the FDA determinations may not be generalizable to other therapeutics given the unique set of regulatory and clinical circumstances that took place during our study time period. Future studies should explore the impact of the sNDA approval process on uptake of a broader range of therapeutics for different indications in the pediatric and other patient populations.

The FDA’s judgments on pediatric supplemental applications were not associated with significant differences in utilization in children and adolescents compared with adult users for the antipsychotic medications in our study. While the agency’s supplemental application process can serve a vital role in the generation of clinical evidence and promotion of evidence-based utilization of therapeutics, better transparency and communication with prescribers of approval and non-approval decisions can help to better optimally translate the FDA’s expertise into clinical practice.

## Supporting Information

S1 Fig Timeline of Important Regulatory and Legal Events for Olanzapine (Zyprexa), Quetiapine (Seroquel), and Ziprasidone (Geodon). (DOCX)

S1 Table ICD-9-CM Codes for Psychiatric Disorders Diagnoses. (DOCX)
