# Supplementary material for: Did FDA Decisionmaking Affect Anti-Psychotic Drug Prescribing in Children?: A Time-Trend Analysis
Source: PLoS One. 2016 Mar 31;11(3):e0152195. doi: 10.1371/journal.pone.0152195 (PMC4816295; doi:10.1371/journal.pone.0152195)
Supplement: S1 Fig — (DOCX) [file pone.0152195.s001.docx]

1996

2000

2004

2008

2010

1996 Olanzapine (Zyprexa) first approved for schizophrenia

1997 Quetiapine (Seroquel) first approved for schizophrenia

2000 Olanzapine approved for bipolar disorder

2001

Ziprasidone (Geodon) first approved for schizophrenia

Jan 2004 Quetiapine approved for bipolar disorder

Aug 2004

Ziprasidone approved for bipolar disorder

Aug 2007

Risperidone approved for pediatric schizophrenia and bipolar disorder

Oct 2007

Aripiprazole approved for pediatric schizophrenia

Feb 2008 Aripiprazole approved for pediatric bipolar disorder

Jan 2009

Eli Lilly settlement for illegal off-label promotion of olanzapine

June 2009

FDA Advisory Committee reviews pediatric use applications of olanzapine, quetiapine, and ziprasidone

Sep 2009

Pfizer settlement for illegal off-label promotion of ziprasidone

Dec 2009

FDA approves olanzapine and quetiapine for pediatric schizophrenia and bipolar disorder

Apr 2010

AstraZeneca settlement for illegal off-label promotion of quetiapine
