# Supplementary material for: Did FDA Decisionmaking Affect Anti-Psychotic Drug Prescribing in Children?: A Time-Trend Analysis
Source: PLoS One. 2016 Mar 31;11(3):e0152195. doi: 10.1371/journal.pone.0152195 (PMC4816295; doi:10.1371/journal.pone.0152195)
Supplement: S1 Table — (DOCX) [file pone.0152195.s002.docx]

**1. PSYCHOTIC DISORDER**

290.8x other specified senile psychotic conditions

290.9x unspecified senile psychotic condition

295.xx schizophrenia

297.xx paranoid states

298.xx other nonorganic psychoses

299.xx psychoses with origin specific to childhood

780.1x hallucinations

**2. BIPOLAR DISORDER**

296.0x manic disorder, single episode

296.1x manic disorder, recurrent episode

296.4x bipolar affective disorder, manic

296.5x bipolar affective disorder, depressed

296.6x bipolar affective disorder, mixed

296.7x bipolar affective disorder, unspecified

296.8x bipolar affective disorder, other and unspecified

296.99 other specified affective psychoses (e.g., mood swings)
